# CD44 Immunohistochemical Expression in Central and Peripheral Parts of Prostatic Adenocarcinoma: An Institutional Study

**DOI:** 10.3390/medicina60122032

**Published:** 2024-12-09

**Authors:** Romano Oguic, Antun Grskovic, Josip Spanjol, Ivana Mikolasevic, Gordana Djordjevic

**Affiliations:** 1 Department of Urology, University Hospital Rijeka, 51000 Rijeka, Croatia; romano.oguic@medri.uniri.hr (R.O.); antun.grskovic@medri.uniri.hr (A.G.); josip.spanjol@medri.uniri.hr (J.S.); 2 Clinical Institute of Oncology and Radiotherapy, University Hospital Centre Rijeka, 51000 Rijeka, Croatia; ivana.mikolasevic@medri.uniri.hr; 3 Department of Pathology and Cytology, University Hospital Center Rijeka, 51000 Rijeka, Croatia

**Keywords:** prostate cancer, CD44s, Gleason score, prostatectomy, tumor periphery

## Abstract

*Background and Objectives*: Prostate cancer is one of the most commonly diagnosed cancers in the male population and the fifth leading cause of cancer death worldwide in men as of 2022. One of the potential biomarkers that can predict the progression of the disease is the transmembrane adhesion molecule CD44s. The aims of this study were to determine the expression of CD44s in prostate cancer in the central tumor mass and in the tumor periphery of the disease and to compare it with the clinicopathological parameters (PSA, Gleason score, surgical margins, and biochemical recurrence of the disease) in patients treated with radical prostatectomy. *Materials and Methods*: The research was randomized retrospectively during the period from 2001 to 2006. Tissue microarrays of 121 archival acinar prostate carcinoma samples were immunohistochemically evaluated for CD44s expression. The immunoexpression was determined semiquantitatively, taking into account the percentage (0 (0–5%), 1 (6–24%), 2 (26–75%), and 3 (76–100%) and intensity of the membranous staining of the tumor cells (0 absent; 1 weak at 400×; 2 intermediates at 100×; 3 strong at 40×) and calculated to obtain a final score (0–3 were regarded as negative; 4–6 were regarded as positive). *Results*: For statistical purposes, we divided the tumors into two categories: Gleason grade group 1 makes up 80.7% and grade group 2, which includes all the remaining Gleason grade groups (out of 2–5), accounts for 19.3% of the tumors. Grade group 1 had the highest incidence of score 4 (positive expression). There were statistically significantly more positive expressions in those tumors with negative prostatectomy margins (chi square: *p* = 0.001; Cramer V: 0.319). There was no correlation between CD44s expression and biochemical recurrence (*p* = 0.218), nor with the preoperative PSA values (*p* = 0.165). In the grade group 1 tumors, the CD44s immunoexpression and status of prostatectomy margin were statistically significantly related with negative margins (*p* = 0.028). An analysis of the expression of CD44s according to the localization in the central part of the tumor mass and on the periphery of the cancer in the group of tumors with a positive margin did not show a significant correlation because the sample was too small. Descriptively, it can be noted that the expression on the periphery was higher, and the central/peripheral expression ratio was higher in favor of the periphery. *Conclusions*: Our results provide insight into the possible value of CD44s expression for predicting the behavior of prostate tumors and the justification of therapy after a prostatectomy. Also hypothetically, they indicate a protective role of CD44s in a group of well-differentiated tumors at the periphery of the tumor mass. Therefore, it is useful to study the CD44s molecule further in this sense.

## 1. Introduction

Prostate cancer (PCa) is the second most frequent cancer worldwide and the most commonly diagnosed cancer in the male population in almost two thirds of the countries in the world. As the fifth leading cause of cancer death in men in 2022 worldwide, prostate cancer remains a major public health problem [1]. In Croatia, according to the latest official updated data of the Croatian Cancer Registry from 2021, there were 2434 new cases of prostate cancer and 805 deaths. The incidence of prostate cancer in 2021 was 130.30/100,000 inhabitants. Data from 2022 show that the survival from prostate cancer in Croatia is below the European average: the five-year survival in Croatia is 71.2%, while the European average is 83.4% [2,3]. Prostate cancer diagnosis is more accurate today. Digital rectal examination, serum prostate specific antigen (PSA), and prostate biopsy under control transrectal ultrasound are the main diagnostic tools. Despite the availability of these methods, at the time of diagnosis, half of the patients had advanced disease [4]. In recent years, the prostate cancer diagnostics, including early tumor detection, tumor characterization, risk stratification, local staging, and image guidance for biopsy and focal therapy, have been improved with the introduction of multiparametric MRI (mpMRI), which avoids underdiagnosis and overtreatment. A biopsy provides insight into the type of tumor, the degree of differentiation using the Gleason grading system regarding the Gleason score or Gleason grade group, and the extent of the disease [5]. During the histological analysis of prostate biopsies, in over 95%, acinar adenocarcinoma is found. Gleason grading system is based upon the microscopic appearance of prostate carcinoma and have significant prognostic value. Gleason system use the sum of the 2 most prevalent histological arhitectural patterns. With regard to malignancy, the Gleason system grades tumors from 1 to 5. Grade 1 refers to good differentiation, while grade 5 refers to poorly differentiated adenocarcinoma. The first number in the score represents a dominant architecture in more than 50% of the tumor. The ISUP (International Society of Urological Pathology) system of classification and grading introduces five groups based on Gleason’s score. Accordingly, a Gleason score of 6 or lower represents the grade 1 group [6]. Studies investigating the behavior of prostate cancer that are focused on prognostic markers can contribute to the development of therapeutic plans or predict the progression of the disease [7]. Classic prognostic factors like the age of the patients, preoperative serum PSA, pathological grade, surgical margin status, invasion of periprostatic tissue, and capsular incision are known to be independent or significant in predicting the clinical recurrence of the tumor [8,9,10,11].

The identification of effective biomarkers has recently become a major focus in PCa research in order to improve disease outcomes and select patients with clinically more aggressive tumors [12]. Exploring different inhibition strategies targeting adhesion molecules contributes significantly to advancing cancer research and developing therapies [13]. Cell adhesion molecules play a key role in normal tissue development and wound healing [14]. In cancer, alterations in cell adhesion enable tumor cells to bypass the normal cellular restriction mechanisms and promote their invasive potential [15]. Adhesion molecules such as integrins, cadherins, and selectins enable cancer cells to bind more efficiently to the extracellular matrix (ECM) [16]. The interaction between cancer cells and the ECM facilitates tumor growth, local invasion, and prepares the cancer cells to enter the metastatic cascade [17]. One of the potential markers that has been receiving considerable attention for years is the transmembrane adhesion molecule CD44. First described in 1983 as a lymphocyte-homing receptor, it exists in several isoforms. These are formed regularly by alternative splicing within the extracellular domain of the receptor or by post-translational modifications resulting in the shorter standard form (CD44s) and variants (CD44v) [18]. Isoforms are overproduced in special conditions such as inflammation, leukocyte activation, and tumorigenesis. Increased expression of the standard molecule CD44s in malignant tumors is associated with increased aggressiveness, as in lymphomas and colorectal cancer, while decreased expression is found in bladder cancer, endometrial cancer, and lung adenocarcinoma [19]. The CD44s expression in a normal prostate changes during differentiation from basal cells to secretory and neuroendocrine populations. The expression of the CD44s form has been observed in basal cells and is retained until differentiation into a neuroendocrine phenotype, while secretory cells differentiate via cells of intermediate-expression CD44s [20]. In prostate carcinoma, CD44s has been involved in proliferation, invasion, migration, and epithelial–mesenchymal transition (EMT) [21]. CD44s is also recognized as a marker of tumor stem cells. These cells play an important role due to their characteristics in cell self-renewal, epithelial–mesenchymal transition, and resistance to apoptosis [22]. Today, studies are attempting to determine the overlapping occurrence of the CD44s molecule and the special characteristics of stem tumor cells, determining this molecule as an early marker of tumor progression [23,24,25]. Also, the mechanisms of the role of the CD44s molecule in radioresistance and possible markers for therapy are being investigated in prostate cancer cell cultures [26]. Recently, this molecule has been the focus of novel therapeutic approaches, including prostate-cancer-stem-cell-targeted therapy [27]. For this purpose, Nishikawa et al., using CD44 as a stem cell marker, reported that higher expression of CD44 on the periphery of the tumor than in the main mass of the tumor is associated with highly invasive glioblastoma, while lower expression of CD44 on the periphery of the tumor is associated with longer survival [28,29]. The aims of this study were to determine and compare the tumor expression of CD44s molecules with clinicopathological parameters such as PSA, Gleason score, surgical margins, and the progression of the disease in patients treated with radical prostatectomy. In the present study, we also determined and compared the tissue expression of this marker in the central tumor mass and in the tumor periphery in a group of tumors with positive surgical margins.

## 2. Materials and Methods

### 2.1. Patients and Tissue Samples

The research was randomized retrospectively during the period from 2001 to 2006. One-hundred-twenty patients with acinar-type adenocarcinoma of the prostate were selected from the archive of medical records at the Clinic for Urology of the Rijeka Clinical Hospital Center, and appropriate clinical data were collected. In the period from 2001 to 2006, following the EAU guidelines, the best candidates for radical prostatectomy were men with Cap in good physical condition and a life expectancy of 10 years (295), disease stages T1–T2 and T3 with unilateral extracapsular infiltration, Gleason score < 8, and PSA values < 20 ng/mL (296). During this period, androgen deprivation treatment (ADT) with LH–RH agonists or surgical castration, in combination with antiandrogens (complete androgen blockade—CAB) or without, was indicated in patients with extracapsular tumor growth or proven lymphatic metastases [30]. For this reason, our examined group of tumors obtained by prostatectomy is predominantly Gleason grade group 1, i.e., Gleason score 5 or 6 (2 + 3; 3 + 3), which today is low-risk cancer and subject to watchful waiting treatment. We considered well-differentiated tumors suitable for studying CD44s as a biomarker whose expression in tumor or at a positive surgical margin would indicate disease progression. During this period, the most common method of radical prostatectomy in our institution was open surgery, and the percentage of positive prostatectomy margins was 36% of prostatectomies (10–40% in the literature) [31]. Only 10–40% of patients develop biochemical disease recurrence, so it is necessary to subclassify positive margins using different biomarkers to identify tumors with higher risk of disease recurrence [32,33].

Two groups of patients were formed: a group of 71 patients having tumors with negative surgical margins and a second group of 49 patients with tumors showing positive surgical margins. From the archival material of the Department of Pathology of the Faculty of Medicine in Rijeka, tumor material from selected patients obtained by radical prostatectomy was reevaluated for construction of tissue microarrays. Representative areas of formalin-fixed, paraffin-embedded tumor tissue were selected on HE slides and marked on appropriate paraffin blocks. From each carcinoma, two tissue cores (1 mm in diameter) were obtained from the central part of tumor and additionally two cores from the periphery of the tumor adjacent to the positive surgical margins only in the group with positive prostatectomy margins. Cores were arrayed in a recipient paraffin block using MTA Booster OI Manual Tissue Arrayer (Alphalyse, Impasse Paul Langevin, 78370 Plaisir—France, France). The resulting blocks were cut into 5 µm sections for immunohistochemistry.

### 2.2. Sample Preparation and Immunohistochemistry

**Figure 1:**
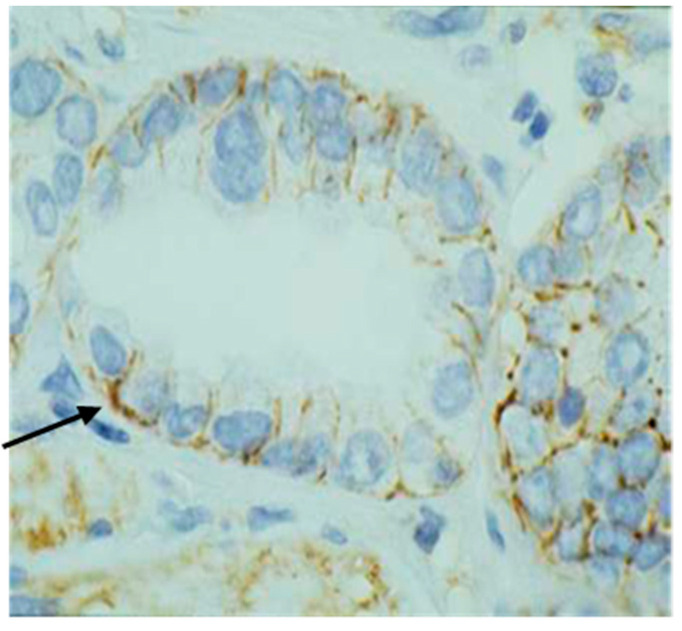
Positive membrane staining (Arrow) for CD44s molecule (magnification 400×), marked as intensity score 1.

**Figure 2:**
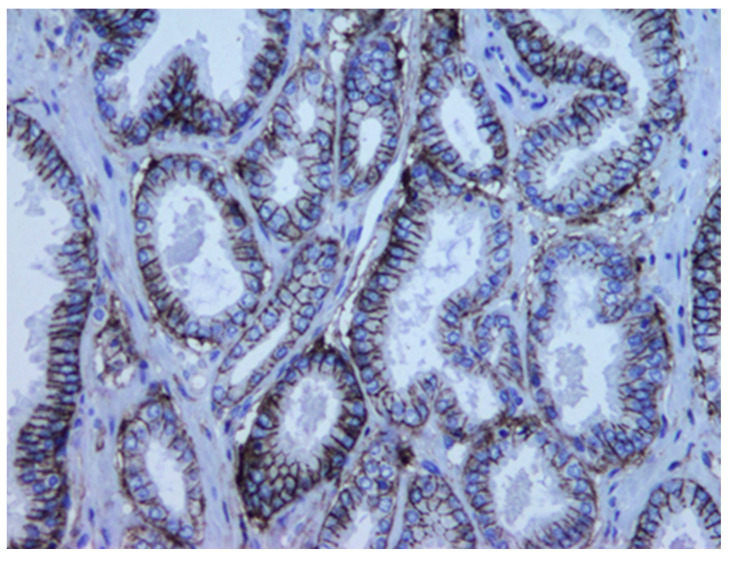
CD44s immunoexpression in well-differentiated PC. Membrane staining is continuous (magnification 200×), labeled as intensity score 2.

**Figure 3:**
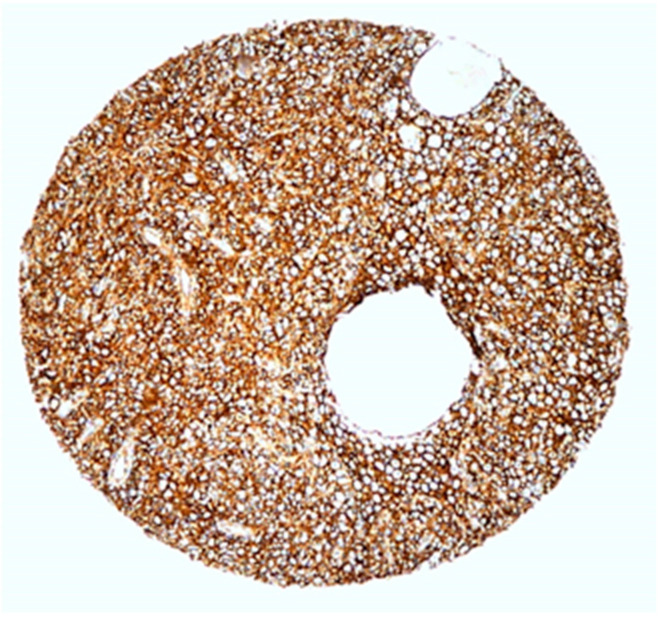
CD44s immunoexpression in poorly differentiated PC. Membrane staining is continuous and strong (magnification 40×), labeled as intensity score 3.

Tissue expression CD44s marker was determined using the standard avidin–biotin immunoperoxidase technique. Immunoreaction to CD44 antibodies (CD44s mouse monoclonal IgG1 antibody clone DF1485, Dakocytomation, Glostrup, Ready to use; Dako Denmark A/Produktionsvej 42,2600 Glostrup Denmark) was evaluated with image analysis system ISSA 3.1 software (Zagreb, Croatia). CD44 expression was evaluated semiquantitatively, taking into account the percentage and intensity of staining of tumor cells and calculated to obtain a final score. Further, 16 tumors could not be adequately evaluated due to technical defects of sample immunostaining, so, finally, 104 tumors were included in the statistical analysis. Immunoexpression of CD44 was membranous, and intensity was graded with 0 (absent), 1 (weak observed at 400× Figure 1), 2 (intermediate observed at 100×, Figure 2), and 3 (strong observed at 40×, Figure 3). The proportions of cell percentage per TMA, positive for CD44, were determined as 0 (0–5%), 1 (6–24%), 2 (26–75%), and 3 (76–100%). A total score of 0–3 was considered negative and 4–6 was considered positive, as described elsewhere in the literature [28,34,35,36]. The expression level of protein was determined with image analysis system ISSA 3.1 software (Zagreb, Croatia).

We have also, separately, evaluated the immunoexpression of the CD44s molecule according to the localization in the central part of the tumor mass and at the periphery of the cancer only in group of tumors with positive resection margins. The staining was evaluated as a percentage of positive tumor cells multiplied by intensity of staining.

### 2.3. Statistical Analysis

The statistical analysis was conducted using standard methods, with a significance level set at *p* < 0.05. Values were represented as number and percentage (%) and median values where applicable. Continuous variables were compared using the Mann–Whitney test. The statistical association between nominal variables was assessed using Cramer’s V coefficient, with a minimum coefficient value of 0.1 required to indicate a meaningful association. Data were processed and presented using MedCalc Statistical Software version 14.8.1.

## 3. Results

### 3.1. Clinicopathological Data

Clinicopathological data were available for 120 patients. The Gleason grade groups, marginal statuses of prostatectomy specimens, biochemical recurrence, pathological stages, and preoperative PSA are shown in Table 1. The majority of the tumors were grade 1 (Gleason score 5 or 6). For statistical purposes, we divided the tumors into two categories: the first category consisted of 96 (80.7%) Gleason grade group 1 tumors. The second category, which included all the remaining Gleason grade groups (2–5), consisted of 23 tumors (19.3%). Biochemical recurrence data were available for 98 patients; the median follow-up time to relapse was 31.2 months in the group of patients with negative prostatectomy margins and 22.6 months in the group of patients with positive margins. 

### 3.2. CD44s Immunoscore in Relation to Gleason Grade Group and Prostatectomy Margins

The CD44 immunoexpression results can be observed in Table 2, shown by score categories from 1–6 and by positive (score > 3)/negative (score < 3) category. The CD44s expression was determined regarding 104 tumors. We included 61 (58.6%) tumors with negative surgical margins and 43 (41.4%) tumors with positive margins.

The grade groups were related to the immunoscore (chi square: *p* = 0.040; Cramer V: 0.336). Grade group 1 had the highest incidence of score 4 (positive expression marked with bold figures), while, in the higher-grade groups, the highest incidence was score 3 (negative expression) (Table 3).

Those tumors with positive margins were related to negative expression; there were statistically significantly more positive expressions (higher scores, mostly 4) in the tumors with negative prostatectomy margins (chi square: *p* = 0.001; Cramer V: 0.319) (Table 4).

In grade group 1, the CD44s immunoexpression and status of prostatectomy margin were statistically significantly related (chi square: *p* = 0.028; Cramer’s V = 0.244). The results are shown in Table 5. Evidently, there were significantly more positive expressions in the tumors with negative margins than in those tumors with positive surgical margins. Within the group of tumors labeled as grade group 2, there was no statistically significant correlation.

There was no correlation between CD44s expression and biochemical recurrence (chi square: *p* = 0.218; Cramer V: 0.133), nor with the preoperative PSA values (the Mann–Whitney test does not show a correlation between PSA level and expression, *p* = 0.165).

### 3.3. CD44s Immunoexpression in Relation to the Periphery and Central Part of the Tumor

Regarding the evaluation of the immunoexpression of CD44s molecules on the peripheries and in the central parts of tumors in a cohort with positive surgical margins, the CD44s expression was calculated as a percentage of positive tumor cells multiplied by intensity of staining.

**Figure 4:**
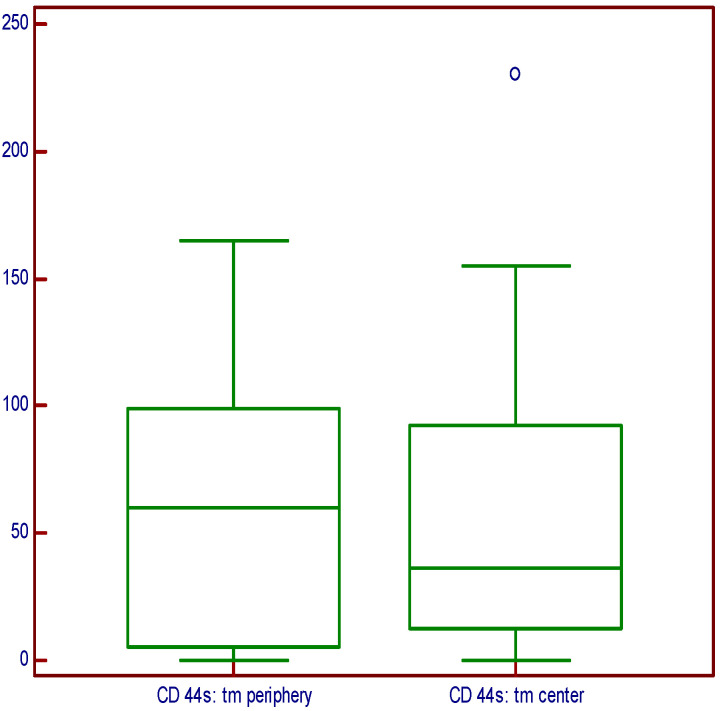
Immunoexpression of the CD44s molecules on the periphery and in the central part of the tumor.

There was no statistically significant difference regarding the expression on the periphery and in the central part of the tumor (*p* = 0.8986), as shown in Figure 4.

**Figure 5:**
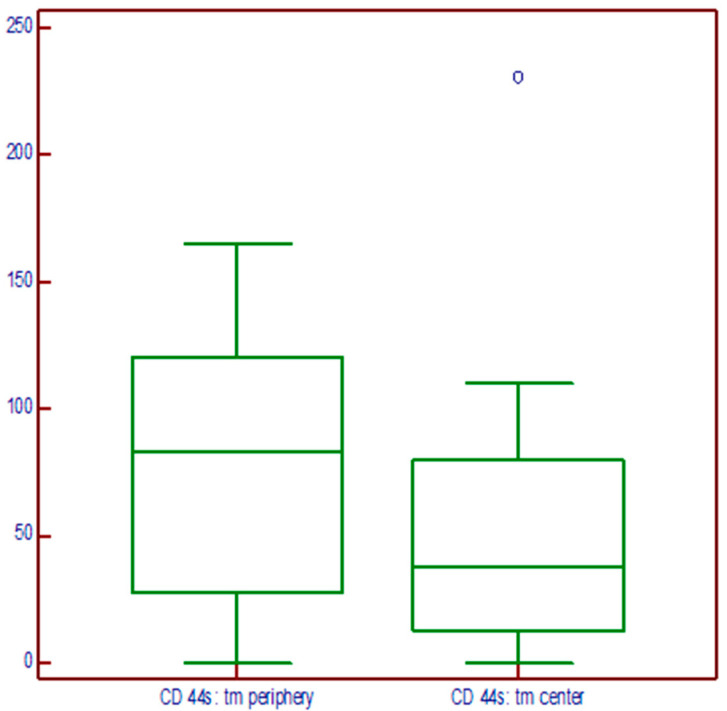
CD44s expression on the periphery and in the central part of the tumors graded as grade group I.

The same results were obtained when we tested the tumors with a positive resection margin within Gleason grade group 1 (*p* = 0.1590). Descriptively, it is evident that the expression at the tumor periphery is higher, but the variability in the measurements was such that there was no statistically significant difference (Figure 5).

**Figure 6:**
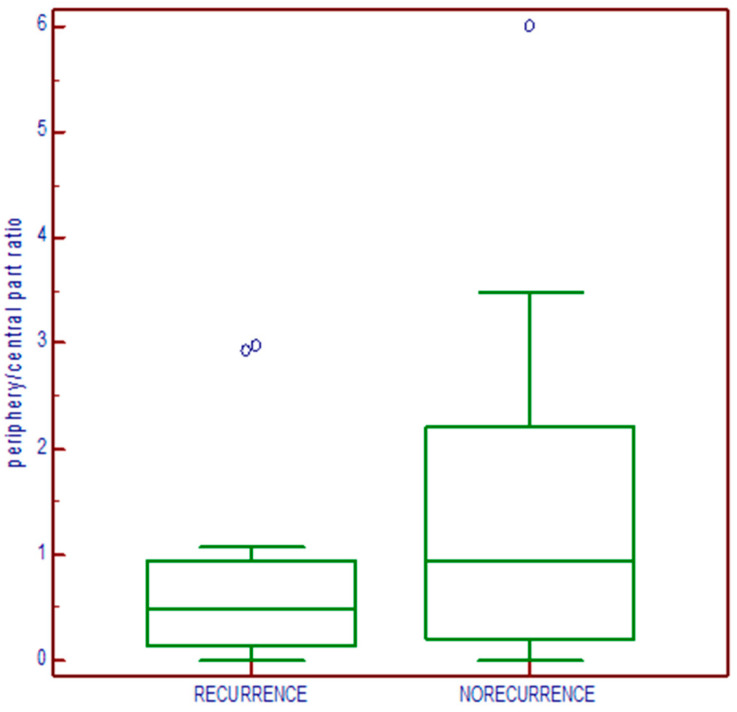
Relationship of CD44s expression shown as the peripheral/central part ratio with disease outcome.

There was no statistical significance (*p* = 0.1087) regarding the relationship with disease outcome when the periphery/central part ratio was tested, although, descriptively, it can be seen that the median of the ratio was higher in those patients without biochemical recurrence (Figure 6). The median ratio in the group without biochemical recurrence was 0.95 (range: 0–6), while, in the group with biochemical recurrence, it was 0.493 (range: 0–2.969).

**Figure 7:**
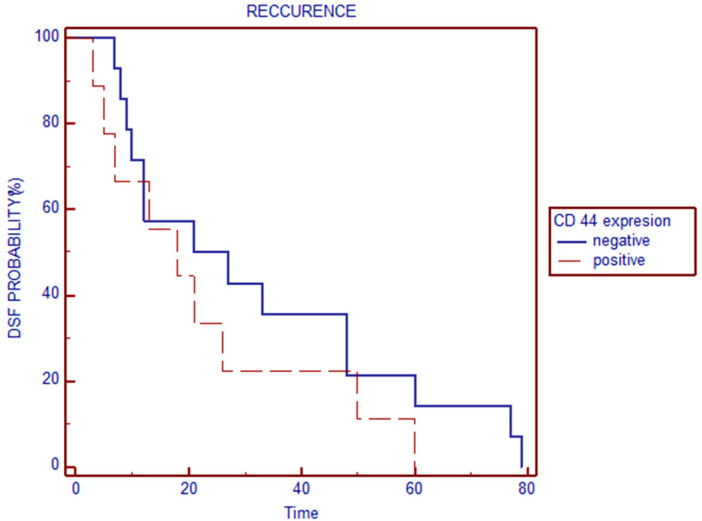
Relationship of CD44s expression shown as the peripheral/central part ratio with disease outcome.

We introduced Kaplan–Meier and log-rank tests (taking into consideration the time course) in analyzing the relation of CD44s immunoexpression to biochemical recurrence. The log-rank test did not show an association between CD44 expression score (negative/positive) and biochemical recurrence (*p* = 0.3095) (Figure 7).

## 4. Discussion

The early detection of prostate cancer with a lower grade, clinically localized, and treated with aggressive therapy, such as prostatectomy, significantly reduces the risk of metastases and symptomatic local tumor growth [34,35]. The classical predictors of clinical and biochemical recurrence, such as preoperative PSA, Gleason grade group, extraprostatic invasion, and positive surgical margins, remain fundamental for access to therapy, but additional information could also be provided by introducing biomarkers as predictive tools. CD44s belongs to a group of transmembrane receptors of extracellular matrix proteins and different growth factor receptors. It is involved in the various mechanisms of cell-to-ECM interaction and therefore enables the migration, differentiation, and activation of cells during embryogenesis, as well as the growth and migration of tumor cells [37]. The CD44 gene undergoes alternative splicing, resulting in the isoforms forming. The interactions with ligands, like hyaluronic acid (HA), osteopontin (OPN), and matrix metalloproteinases (MMPs), connect numerous cancer-associated signaling aspects. The results regarding loss or gain in CD44 expression are controversial in association with clinicopathological features suggesting aggressive tumor characteristics, such as a higher stage, a higher grade, and a lower survival rate [24]. There is evidence regarding the existence of a CD44s-positive subpopulation of prostate tumor stem cells that possess higher proliferative, tumorigenic, and metastatic potential [38]. In relation to grade, our results indicate loss of expression regarding this marker in samples of poorly differentiated tumors, while well-differentiated tumors show continuous and diffuse positive membrane reactions. The literature data indicate the reduced expression of the CD44s molecule in relation to a higher tumor grade. The expression values of CD44s and CD44v6 inversely correlate with tumor grade, stage, and proliferation, which would confirm these molecules as useful prognostic markers of prostate cancer aggressiveness [39]. The immunohistochemical expression of CD44s in radical prostatectomies has been investigated in a few studies with mixed results. An independent correlation with disease progression was obtained by Brewster et al. [40], while Noordzij et al., on 97 radical prostatectomies, found an inverse relationship with the Gleason score, pathological stage, and clinical progression of the tumor [41]. The reduced expression of this marker in a study increased the risk of disease relapse [40]. In our research, no correlation was found between CD44s expression and biochemical recurrence (chi square: *p* = 0.218; Cramer’s V: 0.133), nor with preoperative PSA values (the Mann–Whitney test does not show a correlation between PSA level and expression, *p* = 0.165). Cao and co-authors warned about the problem of positive resection margins in radical prostatectomy and found that the Gleason score determined in the tumor at the resection margin has predictive value regarding the biochemical return of the disease [42], while Alkhateeb S. et al. found that a positive margin is an independent prognostic factor for medium- and high-risk tumors but not for low-risk tumors [43]. A reduced proportion of CD44s-positive cells was associated with vesicle infiltration, positive resection margins with high Gleason scores, and the mitotic index in the study by Aaltomaa et al. [44]. Our study demonstrated that tumors with positive margins were related to negative expression; there were statistically significantly more positive expressions (higher scores, mostly 4) in those tumors with negative prostatectomy margins (chi square: *p* = 0.001; Cramer V: 0.319 s). Our study population included patients with prostate cancer with tumors predominantly graded as Gleason group 1. Large studies of radical prostatectomy (RP) specimens with such tumors showed that they were organ-limited without invasion and metastatic spreading and can be considered to be clinically insignificant [45,46]. Gleason grade group 1 lesions are not fully understood. They may grow in an invasive manner histologically and have some molecular features in common with high-grade cancers, but they do not metastasize. Recently, the discussion regarding these groups of tumors has been renewed. Radiologic and multiple blood and urine tests now explicitly dichotomize high-grade cancer (GG ≥ 2) vs. grade group 1. The characteristics that enable these well-differentiated cancers to spread should be detected [47]. Our examined cohort of tumors obtained by prostatectomy is predominantly of Gleason grade group 1, which we considered to be suitable for studying CD44s as a biomarker, whose expression or loss of expression in tumors or at a positive surgical margin would hypothetically indicate disease progression. The question arose as to whether the CD44s molecules in these tumors can, in a protective manner, contribute to the limitation of the tumors only to the organ area. A recent institutional study from Damarasingu P.V. showed that a loss of positivity in poorly differentiated carcinoma and a loss of CD44 expression in lower tumor staging were associated with greater tumor aggressiveness [29]. Furthermore, a limited number of studies also investigated different expressions regarding CD44 molecules in the central part and in the periphery of the tumor. One of the studies on glioblastoma demonstrated that the periphery/core (P/C) ratio of the CD44 expression in glioblastoma was significantly correlated with responsiveness to the angiogenic inhibitor, Bevacizumab, in the treatment of recurrent tumors [29,48]. Our results demonstrate that, in tumors with a positive surgical margin, there was no statistically significant difference in the expression on the periphery and in the central part of the tumor, but, descriptively, it is evident that, in tumors graded as grade group 1, the expression at the tumor periphery was higher. The variability in the measurement was high, so there was no statistically significant difference. Concerning the periphery/central part ratio, there was no statistical significance in relation to disease outcome. The sample was too small to prove the observation that the median ratio was higher in patients without biochemical recurrence.

## 5. Conclusions

The present study indicates the potential value of CD44s as one of the predictive markers in prostate cancer that is worthy of further research. Indeed, the results point to the protective role of CD44s in a group of well-differentiated tumors at the periphery of the tumor mass. Descriptively, it is evident that the expression at the tumor periphery is higher. Therefore, it is useful to study CD44s molecules further in this regard. Our study has some limitations, such as the relatively small number of analyzed cases and its retrospective design, so larger studies that will prospectively test this biomarker in patients with localized prostate cancer are necessary.

## Figures and Tables

**Table 1:** Clinicopathological data (120 patients).

Grade Group *	N (%)
1	96 (80.7%)
≥2	23 (19.3%)
Margins	
Neg.	71 (59.2%)
Pos.	49 (40.8%)
pT	
pT2	89 (74%)
pT3	31 (26%)
Biochemical recurrence **	
Yes	28 (28.6%)
No	70 (71.4%)
PSA (ng/mL, median)	8.87 ± 7.56

* missing data for grade group for 1 patient; ** Biochemical recurrence data were available for 98 patients.

**Table 2:** CD44 immunoexpression SCORE.

**CD44 immunoexpression SCORE** (immunoexpression determined in 104 tumors)	
1	1 (1%)
2	7 (6.7%)
3	40 (38.4%)
4	48 (46.2%)
5	7 (6.7%)
6	1(1%)
**CD44 immunoexpression SCORE**	
Neg. ≤ 3	48 (46.2%)
Pos. > 3	56 (53.8%)

**Table 3:** CD44s immunoexpression score analysis in relation to the grade group.

		Grade Group		
Score		1	≥2	Total
1	Count % within column	0 0%	1 4.54%	1 0.97%
2		5 6.173%	2 9.09%	7 6.79%
3		26 32.099%	13 59.09%	39 37.84%
4		**42** **51.852%**	6 27.27%	48 46.60%
5		7 8.642%	0 0%	7 8.64%
6		1 1.235%	0 0%	1 0.97%
Total *		81 100%	22 100%	103 100%

Chi square: *p* = 0.040; Cramer V: 0.336; missing data for grading group for one tumor *.

**Table 4:** Analysis in relation to positive/negative prostatectomy margins.

		Pos. Margins		
Score		neg.	pos.	Total
Neg.	Count % within column	20. 32.78%	28 65.11%	48 46.154%
Pos.		41 67.21%	15 15.00%	56.000 53.846%
Total		61 10.00%	43 100.00%	104.000 100.000%

Chi square: *p* = 0.001; Cramer V: 0.319.

**Table 5:** CD44s expression scores regarding tumor grade group I and prostatectomy margin status.

			Positive/Negative Expression		
Grade Group Margins			Neg.	Pos.	Total
1	Neg.	Count % within column	17 30.35%	39 69.64%	56 100.00%
	Pos.		14 56.00%	11 61.72%	25 100.00%
	Total		31 38.27%	50 61.72%	81 100.00%
2	Neg.		2 50.00%	2 50.00%	4 100.00%
	Pos.		14 77.77%	4 22.22%	18 100.00%
	Total		16 72.72%	6 27.27%	22 100.00%
3	Neg.		20 32.78%	41 67.21%	61 10.00%
	Pos.		28 65.11%	15 34.88%	43 100.00%
	Total		48 46.15%	56 53.84%	104 100.00%

Chi square: *p* = 0.028; Cramer V: 0.244.

## Data Availability

The original contributions presented in the study are included in the article, further inquiries can be directed to the corresponding authors.
